# Comparison of aortoiliac disease stenting patency using balloon-expandable, self-expanding, and covered stents

**DOI:** 10.3389/fcvm.2026.1691067

**Published:** 2026-05-13

**Authors:** Ali A. Hasnie, Tonga Nfor, James O. Adefisoye, Kelly Magee, M. Fuad Jan, Mark W. Mewissen, Tanvir K. Bajwa, Babak Haddadian

**Affiliations:** 1 Aurora Cardiovascular and Thoracic Services, Aurora Sinai/Aurora St. Luke’s Medical Centers, Milwaukee, WI, United States; 2 Division of Cardiovascular Medicine, University of Wisconsin School of Medicine and Public Health, Milwaukee Clinical Campus, Milwaukee, WI, United States; 3 Academic Affairs, Aurora UW Medical Group, Aurora Sinai Medical Center, Advocate Aurora Health, Milwaukee, WI, United States; 4 Department of Biostatistics and Data Science, Division of Public Health Sciences, Wake Forest University School of Medicine, Winston-Salem, NC, United States

**Keywords:** aortoiliac stenting, balloon-expandable stent, covered stent, peripheral artery disease, self-expanding stent

## Abstract

**Objective:**

Peripheral arterial disease (PAD) is estimated to affect 6.5 million individuals in the United States. In one-third of cases, PAD is located in the iliac arteries. It has been postulated that covered stents may provide superior outcomes, including patency, compared with other stent types (including balloon-expandable and self-expanding); however, these conclusions have largely been based on a relatively small number of studies. Large studies examining patency rates in real-world populations are lacking. We investigated patency rates of balloon-expandable, self-expanding, and covered stents in PAD using an international database.

**Methods:**

The Vascular Quality Initiative database was queried for all patients undergoing aortoiliac artery disease interventions with covered, balloon-expandable, or self-expanding stents between 2012 and 2017. Of the 7,517 patients identified, 5,631 were excluded for various reasons. Ultimately, 1,886 patients with a total of 2,673 lesions were included for analysis. Baseline demographic data and periprocedural variables were assessed. Primary patency rates were examined at 1 and 3 years.

**Results:**

The majority (58%) of stents implanted were balloon-expandable stents. The overall technical success rate was excellent in all three stent groups (>99%). One-year primary patency rates suggested a small benefit for balloon-expandable stents (98.4%) compared with self-expanding (97.1%) and covered stents (96.3%) (*P* = 0.03); however, this finding did not persist at 3 years (*P* = 0.17).

**Conclusion:**

Contemporary real-world data suggest that there is no meaningful correlation between stent selection and 3-year primary patency in aortoiliac PAD interventions. Stent selection may be best guided by factors such as cost, lesion characteristics, and operator judgment, rather than long-term patency outcomes.

## 1 Introduction

Peripheral arterial disease (PAD) is generally defined as reduced perfusion to the lower extremities, often attributable to atherosclerotic vascular disease. It is typically diagnosed by an ankle–brachial index <0.90. Patients with PAD may present with intermittent claudication, which may progress to critical limb ischemia. True estimates of PAD remain elusive, owing in part to varying definitions of PAD and varying methods of detection. Prevalence ranges from 5.8% to 10.7% based on data collected prior to 2010 (1–3). Population-based estimates indicate that about 6.5 million people >40 years of age in the USA have PAD based on an ankle–brachial index <0.9; if false negative tests are included, that estimate increases to 8.5 million individuals (1). The incidence of PAD will likely continue to grow as US demographics shift and the baby boomer population ages.

Nearly 30% of the arterial lesions in PAD are located in the iliac arteries (4). Endovascular intervention of the aortoiliac arteries was introduced in 1964 (5), and today an endovascular-first approach is increasingly considered the standard of care. However, considerable variability exists in stent selection for the treatment of aortoiliac artery disease.

Each type of stent offers distinct advantages and limitations. Balloon-expandable stents (BESs) offer enhanced radial strength and precise deployment, but are limited by the need for larger sheath sizes. Self-expanding stents (SESs) require smaller sheaths and generally offer longer lengths, but are limited by lesser radial strength and precision. Covered stents (CSs) have primarily been utilized for iatrogenic perforations or iliac artery aneurysms. Stent selection has largely been guided by opinion because of the lack of large studies providing clear indications. Given the conflicting data regarding stent selection in aortoiliac artery disease, we sought to use a large, real-world database to compare the primary patency rates of BES, SES, and CS.

## 2 Methods

### 2.1 Dataset

This registry-based retrospective study utilized the Vascular Quality Initiative (VQI) database, a large national database that collects more than 200 patient- and procedure-specific variables, as well as in-hospital outcomes data, from more than 450 centers and 3,000 physicians across the USA and Canada. The VQI Committee approved the study protocol (Project ID 3940), and the local institutional review board determined that the project did not constitute human subject research. Patient consent was not required. AH had full access to the data and assumes full responsibility for the integrity of the data and statistical analyses.

### 2.2 Inclusion and exclusion criteria

The VQI database was queried for all patients who had undergone aortoiliac intervention between January 2012 and September 2017. Patients who had had a prior intervention and those who had acute limb ischemia were excluded, as were those for whom treatment type, stent type, or another key variable was missing. Of the 7,517 patients identified, 5,631 patients were excluded and 1,886 patients with a total of 2,673 lesions were included (Supplementary Figure 1). Each lesion received at least one stent: 62.5% (*n* = 1,179) of the lesions received one stent, 34.3% received two stents, 2.1% received three stents, and 1.1% received four stents. For the purpose of analyzing patient characteristics/demographics, as provided in Table 1, patients were grouped based on either the first or the only stent they received. Lesion-level data, as provided in Table 2, were grouped by stent type. Baseline demographic data and periprocedural variables were assessed. Primary patency rates were assessed for all stent types.

**Table 1:** Patient demographics.

Demographic variable	Total (*n* = 1,886)	Covered stents (*n* = 377)	Balloon-expandable stents (*n* = 1,079)	Self-expanding stents (*n* = 430)	*P*-value
Age, years	65.0 (58.0–72.0)	66.0 (58.0–73.0)	65.0 (58.0–72.0)	65.0 (57.0-72.0)	0.35
Male sex	1,038 (55.0)	209 (55.4)	565 (52.4)	264 (61.4)	0.01
Race: non-White	220 (11.7)	39 (10.3)	121 (11.2)	60 (14.0)	0.22
Height, cm	170.0 (162.0–176.0)	170.0 (160.0–176.0)	168.0 (160.0–177.0)	170.0 (163.0–175.0)	0.45
Weight, kg	78.0 (65.0–91.0)	77.2 (66.0–89.0)	79.0 (66.0–91.0)	76.0 (65.0–90.0)	0.29
BMI	27.1 (23.6–30.8)	27.0 (23.7–30.9)	27.3 (24.0–31.0)	26.6 (23.1–30.0)	0.04
CAD	507 (26.9)	108 (28.7)	286 (26.5)	113 (26.3)	0.69
CHF	171 (9.1)	38 (10.1)	99 (9.2)	34 (7.9)	0.55
COPD	514 (27.3)	112 (29.7)	284 (26.3)	118 (27.4)	0.44
Diabetes	678 (36.0)	127 (33.7)	390 (36.1)	161 (37.4)	0.53
Dialysis	16 (0.9)	2 (0.5)	11 (1.0)	3 (0.7)	0.62
Hypertension	1,557 (82.6)	299 (79.3)	887 (82.2)	371 (86.3)	0.03
Smoking	1,748 (92.7)	346 (91.8)	999 (92.6)	403 (93.7)	0.56
Pre-ACE/ARB	931 (49.4)	184 (48.8)	514 (47.6)	233 (54.2)	0.07
Pre-ASA	1,384 (73.4)	285 (75.6)	773 (71.6)	326 (75.8)	0.14
Pre-anticoagulation	169 (9.0)	45 (11.9)	83 (7.7)	41 (9.5)	0.04
Pre-antiplatelet–P2Y12 inhibitor	634 (33.6)	109 (28.9)	367 (34.0)	158 (36.7)	0.06
Pre-statin	1,346 (71.4)	274 (72.7)	757 (70.2)	315 (73.3)	0.40
Preop leg symptoms—right					
Asymptomatic	390 (20.7)	97 (25.7)	193 (17.9)	100 (23.3)	<0.01
CLI	318 (16.9)	77 (20.4)	168 (15.6)	73 (17.0)	0.10
Claudication	940 (49.8)	170 (45.1)	577 (53.5)	193 (44.9)	<0.01
Not treated	158 (8.4)	13 (3.5)	104 (9.6)	41 (9.5)	<0.01
Preop leg symptoms—left					
Asymptomatic	371 (19.7)	100 (26.5)	183 (17.0)	88 (20.5)	<0.01
Claudication	966 (51.2)	184 (48.8)	572 (53.0)	210 (48.8)	0.20
CLI	321 (17.0)	68 (18.0)	179 (16.6)	74 (17.2)	0.81
Not treated	146 (7.7)	12 (3.2)	98 (9.1)	36 (8.4)	<0.01
Target lesion dissection^a^	*n* = 161 1 (0.6)	*n* = 161 1 (0.6)	*n* = 0	*n* = 0	—
Preop creatinine^a^, mg/dL	*n* = 1,834 0.9 (0.8–1.1)	*n* = 368 0.9 (0.8–1.1)	*n* = 1,052 0.9 (0.8–1.1)	*n* = 414 0.9 (0.8–1.2)	0.40
Preop ABI-right^a^	*n* = 1,436 0.7 (0.5–0.9)	*n* = 285 0.7 (0.5–0.9)	*n* = 835 0.7 (0.5–0.9)	*n* = 316 0.7 (0.5–0.9)	<0.01
Preop ABI-left^a^	*n* = 1,435 0.7 (0.5–0.9)	*n* = 283 0.6 (0.5–0.9)	*n* = 832 0.7 (0.5–0.9)	*n* = 320 0.7 (0.5–0.9)	<0.01
Fluoroscopy time^a^	*n* = 1,742 7.9 (4.7–13.4)	*n* = 345 8.4 (5.0–16.1)	*n* = 1,006 7.0 (4.3–12.0)	*n* = 391 9.5 (5.8–15.0)	<0.01
Contrast volume^a^	*n* = 1,823 70.0 (42.0–115.0)	*n* = 361 68.0 (40.0–105.0)	*n* = 1,042 70.0 (42.0–115.0)	*n* = 420 75.0 (45.0–120.0)	0.02

Data presented as *n* (%) or median (IQR). ABI, ankle–brachial index; ACE, angiotensin-converting enzyme; ARB, angiotensin receptor blocker; ASA, acetylsalicylic acid; BMI, body mass index; CAD, coronary artery disease; CHF, congestive heart failure; CLI, critical limb ischemia; COPD, chronic obstructive pulmonary disease; IQR, interquartile range (Q1-Q3); Pre, premedication; Preop, preoperative.

a Number of available data (*n*=) displayed next to statistics for variables with missing data. *P*-values based on chi-square or Kruskal–Wallis test.

**Table 2:** Lesion characteristics.

Lesion variable	Total (*n* = 2,673)	Covered stents (*n* = 578)	Balloon-expandable stents (*n* = 1,518)	Self-expanding stents (*n* = 577)	*P*-value
Artery treated					
Common + external iliac	12 (0.5)	12 (2.1)	0 (0)	0 (0)	<0.01
Common iliac	2,005 (75.0)	425 (73.5)	1,275 (84.0)	305 (52.9)	<0.01
External iliac	656 (24.5)	141 (24.4)	243 (16.0)	272 (47.1)	<0.01
Final technical result—successful (stenosis ≤30%)	2,668 (99.8)	577 (99.8)	1,515 (99.8)	576 (99.8)	1.00
Side of procedure—left	1,341 (50.2)	285 (49.3)	765 (50.4)	291 (50.4)	0.90
TASC grade	*n* = 2,348	*n* = 509	*n* = 1,338	*n* = 501	
A	1,170 (49.8)	167 (32.8)	774 (57.9)	229 (45.7)	<0.01
B	568 (24.2)	128 (25.2)	301 (22.5)	139 (27.7)	0.09
C	302 (12.9)	92 (18.1)	129 (9.6)	81 (16.2)	<0.01
D	35 (1.5)	35 (6.9)	0 (0)	0 (0)	<0.01
Protect adjacent artery	273 (11.6)	87 (17.1)	134 (10.0)	52 (10.4)	<0.01
Calcification^a^	*n* = 240	*n* = 240	*n* = 0	*n* = 0	—
Focal/mild	51 (21.3)	51 (21.3)	—	—	—
Moderate	55 (22.9)	55 (22.9)	—	—	—
None	81 (33.8)	81 (33.8)	—	—	—
Not evaluated	9 (3.8)	9 (3.8)	—	—	—
Severe	44 (18.3)	44 (18.3)	—	—	—
Stent size/diameter^a^, mm	*n* = 2,566 8.0 (7.0–9.0)	*n* = 501 8.0 (7.0–9.0)	*n* = 1,499 8.0 (7.0–8.0)	*n* = 566 8.0 (7.0–9.0)	<0.01
Treated length^a^	*n* = 188 40.0 (38.0–59.0)	*n* = 188 40.0 (38.0–59.0)	*n* = 0	*n* = 0	—

Data presented as *n* (%) or median (interquartile range). TASC, Trans-Atlantic Inter-Society Consensus Document on Management of Peripheral Arterial Disease.

a Number of available data (*n*=) displayed next to statistics for variables with missing data. *P*-values based on chi-square, Fisher's exact, or Kruskal–Wallis test.

### 2.3 Variable definitions

Variable definitions followed those provided in the VQI database (Supplementary Material).

### 2.4 Outcomes

The primary outcome of interest was the primary patency rate, measured at 1 and 3 years. Patency was assessed utilizing the ankle–brachial index (increase <0.15), evidence of angiographic stenosis, duplex ultrasound, or magnetic resonance angiography. Secondary outcomes included the rates of amputation and target lesion revascularization.

### 2.5 Statistical analysis

Categorical variables were presented as frequency and percentage; continuous variables were assessed for normality using the Shapiro–Wilk test and presented as median [interquartile range (IQR)] owing to non-normality. The association between categorical variables was assessed using Pearson's chi-square or Fisher's exact test, as appropriate, and comparisons of groups of continuous variables were carried out using the Kruskal–Wallis test. The primary outcome of 1-year and 3-year patency rates was tested for difference between the three stent types using the log-rank test and visualized with Kaplan–Meier curves. Univariable and multivariable Cox proportional hazards regression models were developed to determine factors independently associated with the primary patency of the deployed stents at 1 year. These variables were then entered into a multivariable model. Hazard ratios and adjusted hazard ratios along with 95% confidence interval (CI) were reported as appropriate. Where necessary, as in the case of a small sample size owing to missing data or symptom monotonicity, Firth's penalized likelihood method was used to obtain a finite estimate for the coefficients. For all analyses, *P*-values ≤0.05 were considered statistically significant. Statistical analyses were performed using SAS 9.4 (SAS Institute, Cary, NC, USA).

## 3 Results

A total of 1,886 patients with 2,673 unique lesions were included. Of these, 377 (20.0%) were in the CS group, 1,079 (57.2%) in the BES group, and 430 (22.8%) in the SES group. The median age of the patients was 78 years (IQR, 68–84 years). In all groups, male sex and White race/ethnicity were predominant. A substantial proportion of patients had multiple comorbidities. The majority of patients required intervention because of claudication (49.8%, right leg; 51.2%, left leg). The average baseline ankle–brachial index was generally <0.9 in both the right and left lower extremities across all groups, suggesting significant atherosclerotic disease in the cohort (Table 1).

Procedural characteristics varied across stent groups. The BES group had the lowest fluoroscopic time (median 7.0 min, IQR 7.3–12 min), followed by the SES group [8.4 (5.0–16.1) min] and then the CS group [9.5 (5.8–15.0) min] (*P* < 0.01). The CS group required 68 mL (40–105 mL) of contrast volume for deployment, which was significantly less than the BES group [70 (42–115) mL] and SES group [75 (45–120) mL] (*P* = 0.02) (Table 1).

Although distribution varied, the majority of interventions occurred in the common iliac arteries (CS 73.5%, BES 84.0%, SES 52.9%, *P* < 0.01) followed by the external iliac arteries (CS 24.4%, BES 16.0%, SES 47.1%, *P* < 0.01) in all groups. Overall technical success was excellent across all groups (CS 99.8%, BES 99.8%, SES 99.8%, *P* > 0.99). The majority of intervened lesions were classified as either TASC A or TASC B (CS 58.0%, BES 80.4%, and SES 73.4%). Although calcification data were not available for all stent types, the CS group demonstrated that about 40% (99/240) of the stents were deployed in moderate to severely calcified vessels (Table 2).

Generally, in all three groups, no more than two arteries were stented during the index procedure. Thirty-day mortality rates remained low and were not significantly different between groups (CS 7.2%, BES 9.6%, SES 10.7%, *P* = 0.21). Patients remained largely asymptomatic at follow-up, with no differences noted between the left and right lower extremities. Amputation rates were low across all groups, with no significant differences noted (Table 3).

**Table 3:** Patient post-procedure variables.

Post-procedure variable	Total (*n* = 1,886)	Covered stents (*n* = 377)	Balloon-expandable stents (*n* = 1,079)	Self-expanding stents (*n* = 430)	*P*-value
Number of arteries treated					
1	1,179 (62.5)	202 (53.6)	672 (62.3)	305 (70.9)	<0.01
2	647 (34.3)	156 (41.4)	384 (35.6)	107 (24.9)	<0.01
3	40 (2.1)	12 (3.2)	14 (1.3)	14 (3.3)	0.02
4	20 (1.1)	7 (1.9)	9 (0.8)	4 (0.9)	0.26
Deceased	177 (9.4)	27 (7.2)	104 (9.6)	46 (10.7)	0.21
ACE/ARB	900 (47.7)	180 (47.8)	511 (47.4)	209 (48.6)	0.91
ASA	1,517 (80.4)	312 (82.8)	846 (78.4)	359 (83.5)	0.04
P2Y12 antagonist–P2Y12 inhibitor	989 (52.4)	186 (49.3)	563 (52.2)	240 (55.8)	0.18
Anticoagulation	286 (15.2)	70 (18.6)	137 (12.7)	79 (18.4)	<0.01
Statin	1,498 (79.4)	306 (81.2)	854 (79.2)	338 (78.6)	0.63
Cilostazol	24 (1.3)	12 (3.2)	9 (0.8)	3 (0.7)	<0.01
Leg symptoms—right^a^	*n* = 1,659	*n* = 303	*n* = 961	*n* = 395	
Asymptomatic	1,231 (65.3)	231 (76.2)	712 (74.1)	288 (72.9)	0.18
Claudication	330 (17.5)	54 (17.8)	193 (20.1)	83 (21.0)	0.16
CLI	98 (5.2)	18 (5.9)	56 (5.8)	24 (6.1)	0.88
Leg symptoms—left^a^	*n* = 1,692	*n* = 331	*n* = 969	*n* = 392	
Asymptomatic	1,255 (74.2)	257 (77.6)	719 (74.2)	279 (71.2)	0.61
Claudication	345 (20.4)	59 (17.8)	202 (20.9)	84 (21.4)	0.31
CLI	92 (5.4)	15 (4.5)	48 (5.0)	29 (7.4)	0.12
Amputation—right^a^	*n* = 1,816 51 (2.8)	*n* = 371 7 (1.9)	*n* = 1,036 33 (3.2)	*n* = 409 13 (3.2)	0.46
Amputation—left^a^	*n* = 1,810 52 (2.9)	*n* = 368 10 (2.7)	*n* = 1,030 33 (3.2)	*n* = 412 9 (2.2)	0.58
ABI—right^a^	*n* = 1,465 0.9 (0.7–1.0)	*n* = 311 0.9 (0.7–1.1)	*n* = 838 0.9 (0.7–1.0)	*n* = 316 0.9 (0.7–1.0)	0.03
ABI—left^a^	*n* = 1,467 0.9 (0.7–1.0)	*n* = 310 0.9 (0.7–1.0)	*n* = 831 0.9 (0.7–1.0)	*n* = 326 0.9 (0.6–1.0)	<0.01
Toe pressure—right^a^, mmHg	*n* = 137 40.0 (0.7–88.0)	*n* = 72 41.0 (0.7–88.0)	*n* = 55 47.0 (0.6–98.0)	*n* = 10 27.0 (0.6–70.0)	0.80
Toe pressure—left^a^, mmHg	*n* = 140 42.5 (0.7–81.0)	*n* = 72 51.5 (0.7–83.0)	*n* = 56 34.0 (0.6–82.5)	*n* = 12 42.0 (9.4–65.5)	0.61

Data presented as *n* (%) or median (interquartile range). ABI, ankle–brachial index; ACE, angiotensin-converting enzyme; ARB, angiotensin receptor blocker; ASA, acetylsalicylic acid; CLI, critical limb ischemia.

a Number of available data (*n*=) displayed next to statistics for variables with missing data. *P*-values based on chi-square, Fisher's exact, or Kruskal–Wallis test.

**Figure 1:**
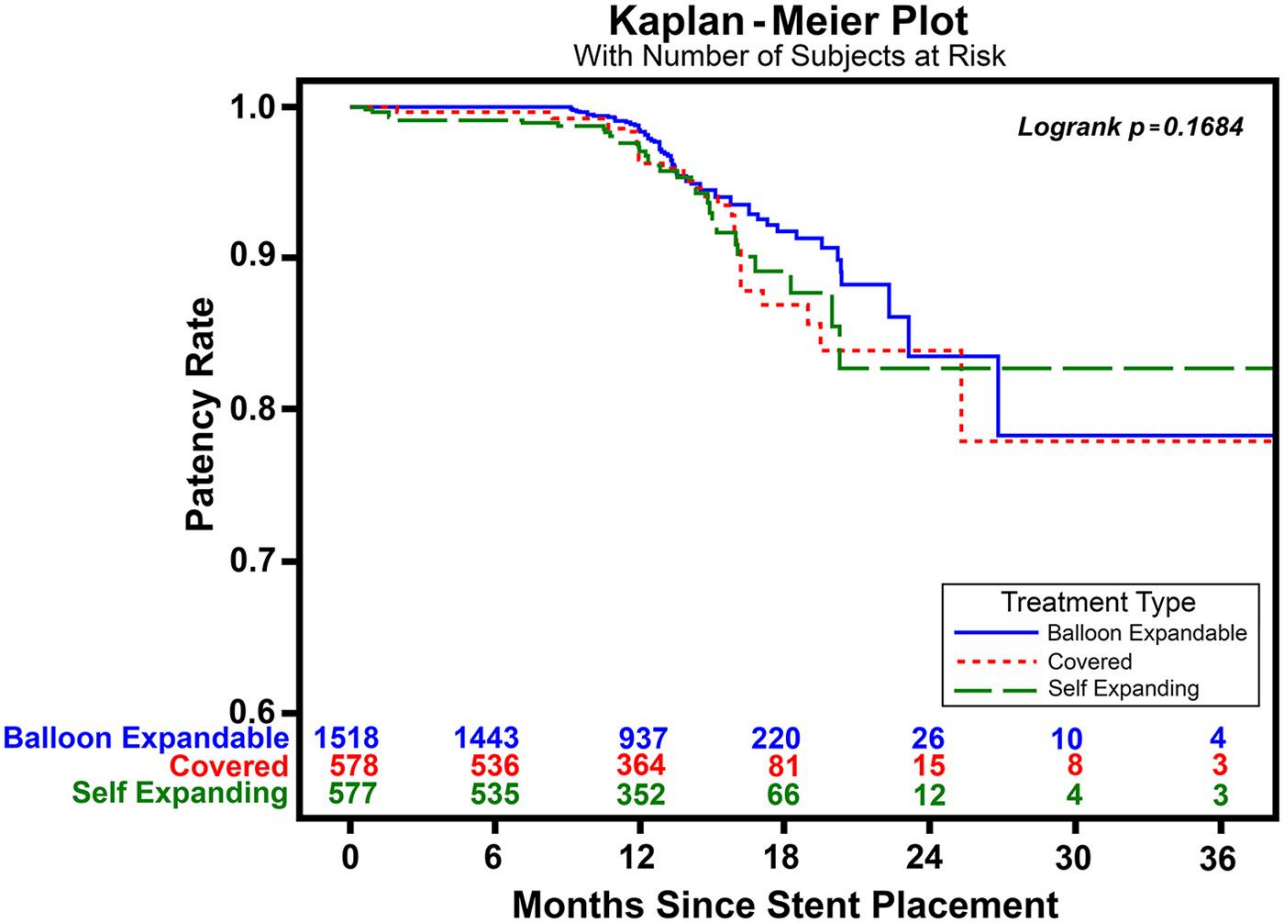
One-year patency Kaplan–Meier curve. Primary patency rates remained high for all stent types at 1 year, with BESs appearing most favorable.

Regarding the primary outcomes, the 1-year primary patency rate was high (>95%) in all groups (Figure 1), but most favorable in the BES group (CS 96.3%, BES 98.4%, SES 97.1%, *P* = 0.03). Given the high rate of initial success, it was unsurprising that the reintervention rate was quite low across groups, with a slightly higher rate observed in the BES group (CS 10.1%, BES 12.2%, SES 10.9%, *P* = 0.36; Table 4). At 3 years, patency rates suggested no significant differences between stent groups (CS 77.9%, BES 78.3%, SES 82.7%, *P* = 0.17; Figure 1). The significant difference noted at 1 year likely disappeared at the 3-year evaluation owing to the larger sample size noted at 1 year and the significant loss of patient follow-up at 3 years.

**Table 4:** All-time primary patency and target lesion revascularization: follow-up data.

Patency variable	Total (*n* = 2,673)	Covered stents (*n* = 578)	Balloon-expandable stents (*n* = 1,518)	Self-expanding stents (*n* = 577)	*P*-value
Patency	2,545 (95.2)	544 (94.1)	1,455 (95.9)	546 (94.6)	0.19
Patency judged by					
ABI increase	425 (15.9)	93 (16.1)	246 (16.2)	86 (14.9)	0.76
Angiography	195 (7.3)	23 (4.0)	115 (7.6)	57 (9.9)	<0.01
CTA	233 (8.7)	76 (13.2)	96 (6.3)	61 (10.6)	<0.01
Duplex ultrasound	1,799 (67.3)	385 (66.6)	1,045 (68.8)	369 (64.0)	0.10
MRA	21 (0.8)	1 (0.2)	16 (1.1)	4 (0.7)	0.12
Percent stenosis <50%	*n* = 477 408 (85.5)	*n* = 237 206 (86.9)	*n* = 185 157 (84.9)	*n* = 55 45 (81.8)	0.59
Target lesion intervention	*n* = 45 35 (77.8)	*n* = 17 16 (94.1)	*n* = 20 13 (65.0)	*n* = 8 6 (75.0)	0.10
Retreatment for thrombosis	*n* = 489 17 (3.5)	*n* = 283 9 (3.2)	*n* = 159 7 (4.4)	*n* = 47 1 (2.1)	0.69
Artery retreatment	*n* = 2,648	*n* = 576	*n* = 1,499	*n* = 573	
Interventional	191 (7.2)	34 (5.9)	121 (8.1)	36 (6.3)	0.14
Surgical	113 (4.2)	24 (4.2)	62 (4.1)	27 (4.7)	0.84
None	2,344 (88.5)	518 (89.9)	1,316 (87.8)	510 (89.1)	0.36

Data presented as *n* (%). ABI, ankle–brachial index; CTA, computed tomography angiography; MRA, magnetic resonance angiography.

a *P*-values based on chi-square or Fisher's exact test.

Given the significant difference observed in the 1-year primary patency rate between stent types, Cox regression models were employed to determine independently associated factors. Two continuous variables (age and body mass index), 10 categorical variables (sex, race, coronary artery disease, congestive heart failure, chronic obstructive pulmonary disease, diabetes, end-stage renal disease on dialysis, hypertension, smoking, and stent type), and two ordinal variables (artery treated and TASC grade) were used to build univariable models for patency. Age, hypertension, TASC grades A, B, and C, and stent type were associated with likelihood of patency at 1 year. These variables were then entered into a multivariable model, from which the only predictors of 1-year primary patency were age, stent type, and TASC grades A, B, and C (Table 5).

**Table 5:** Univariable and multivariable Cox regression analysis of 1-year primary patency.

Variable	Univariable analysis			Multivariable analysis		
	Hazard ratio	95% CI	*P*-value	Hazard ratio	95% CI	*P*-value
Age	0.95	0.93–0.97	<0.01	0.95	0.93–0.98	<0.01
Sex						
Female	Ref	Ref	Ref	Ref	Ref	Ref
Male	0.66	0.38–1.16	0.15	0.76	0.40–1.47	0.42
Race						
Non-white	Ref	Ref	Ref	Ref	Ref	Ref
White	0.77	0.35–1.71	0.52	0.70	0.28–1.71	0.43
BMI	1.00	0.95–1.05	0.98	0.99	0.93–1.05	0.64
Coronary artery disease	1.23	0.65–2.21	0.51	1.91	0.92–3.95	0.08
Congestive heart failure	0.11	0.01–1.75	0.12	0.15	0.01–2.43	0.18
COPD	0.52	0.24–1.11	0.09	0.53	0.22–1.26	0.15
Diabetes mellitus	1.11	0.62–1.98	0.73	1.48	0.75–2.95	0.26
ESRD on hemodialysis	1.14	0.07–19.06	0.93	1.27	0.07–22.47	0.87
Hypertension	0.53	0.29–0.99	0.04	0.49	0.23–1.03	0.06
Smoking	1.75	0.43–7.21	0.44	1.61	0.40–6.44	0.50
Artery treated						
Common iliac	0.79	0.43–1.47	0.46	0.92	0.42–2.01	0.83
External iliac	Ref	Ref	Ref	Ref	Ref	Ref
TASC grade						
A	0.45	0.22–0.905	0.03	0.46	0.22–0.95	0.04
B	0.08	0.02–0.37	<0.01	0.09	0.02–0.35	<0.01
C	0.24	0.07–0.86	0.03	0.26	0.08–0.88	0.03
D	1.26	0.28–5.62	0.76	1.78	0.36–8.67	0.48
Protect adjacent artery	Ref	Ref	Ref	Ref	Ref	Ref
Stent size/diameter	0.95	0.78–1.14	0.57	0.99	0.84–1.16	0.88
Stent type						
Covered	1.13	0.55–2.31	0.74	0.80	0.33–1.97	0.63
Balloon-expandable	0.50	0.25–1.00	0.05	0.45	0.20–0.98	0.04
Self-expanding	Ref	Ref	Ref	Ref	Ref	Ref

BMI, body mass index; CI, confidence interval; COPD, chronic obstructive pulmonary disease; ESRD, end-stage renal disease; Ref, reference level. Analysis was based on complete cases. Twelve patients with common + external iliac artery treated excluded from Cox regression analysis due to small group.

## 4 Discussion

This study represents one of the largest to compare CSs, BESs, and SESs for the treatment of aortoiliac disease. The primary endpoint was the primary patency rate at both 1 and 3 years. One-year primary patency rates were excellent in all stent types and suggested a benefit for BESs; however, 3-year primary patency rates suggested no significant differences between stent types.

The role of endovascular therapy has expanded in recent years, and this treatment modality has demonstrated utility for the management of both simple and complex lesions. Endovascular therapy offers a less invasive option for the management of PAD. Its main limitation was thought to be inferior primary patency rates compared with surgical intervention. This concern has been largely put to rest by studies that found comparable outcomes between surgical bypass and endovascular therapy for up to 3 years of follow-up (6–8). Endovascular approaches also provide lower morbidity than aortobifemoral bypass, with rates ranging from 1% to 15% and infection rates ranging from 0.5% to 5% (9, 10). Surgical intervention morbidity and mortality rates are known to approach 4.4% (11).

Stent selection has largely been guided by operator preference, owing to the lack of clear guidelines and conflicting literature on optimal stent type selection. Traditionally, the Trans-Atlantic Inter-Society Consensus (TASC) recommended endovascular intervention for TASC A/B lesions and open surgical repair for TASC C/D lesions (12). However, recent studies have demonstrated comparable outcomes between these revascularization modalities across all TASC lesion types (13–15).

Support for the use of CSs in aortoiliac artery disease has been largely based on small retrospective studies and an industry-sponsored randomized controlled trial. One of the retrospective studies evaluated 54 patients, which included a greater percentage of patients with TASC C/D lesions being treated with CSs (38.0 vs. 7.0%). The study revealed a statistically significant difference between the patency rates of CSs and bare metal stents (92% vs. 72%) at 2 years of follow-up (16). The randomized controlled Covered Versus Balloon Expandable Stent Trial (COBEST) evaluated the entire aortoiliac segment, with the primary outcomes of binary restenosis and target vessel revascularization (17, 18). This study also suggested that CSs were superior to bare metal stents in TASC C/D lesions. However, subsequent studies have failed to confirm this finding. The DISCOVER (Dutch Iliac Stent trial: COVERed balloon-expandable vs. uncovered balloon-expandable stents in the common iliac artery) trial evaluated lesions >3 cm (19). The investigators found that both stent types showed excellent patency results periprocedurally and at 2 years of follow-up. A more recent systematic review and meta-analysis examining a total of 1,617 patients suggested that TASC D lesions treated with covered stents demonstrated improved primary patency as compared with bare metal stents. Interestingly, this finding did not persist when all TASC subtypes were evaluated (20). This may reflect the fact that experienced operators generally intervene on high-grade TASC lesions; however, further studies are needed to confirm this finding.

The theoretical benefit of a CS lies in its ability to form a mechanical barrier and thus exclude plaque. It is also thought that the polytetrafluoroethylene (PTFE) covering prevents macrophages from migrating through the stent struts and causing an inflammatory cascade and neointimal hyperplasia. Clinically, however, there is no definitive evidence of superiority of CSs compared with bare metal stents. Covered stents appear to require less contrast volume for deployment than balloon-expandable and self-expanding stents, suggesting they may be of benefit in cases in which limitations of contrast administration exist.

SESs and BESs have also been extensively evaluated. A multicenter, retrospective study examining 2,147 patients found similar patency rates in both stent types (BES 79%, SES 75%) (21). The Efficacy Study of Iliac Stents to Treat TASC A-B-C-D Iliac Artery Lesions (BRAVISSIMO) trial, which included experienced operators, reported similar results (22, 23), with no differences in stent type within the four TASC classifications. The Iliac, Common, and External (ICE) Artery Stent Trial—a randomized clinical trial of 660 patients comparing BESs and SESs—found SESs to have a lower binary restenosis rate than BESs (6.1% vs. 14.9%, *P* = 0.006) as well as lower rates of target vessel revascularization at 12 months (24). A more recent randomized controlled trial in South Korea comparing balloon-expandable and self-expanding stents for iliac artery disease found no difference in 1-year outcomes for primary patency (99% vs. 99%, *P* > 0.999) (25).

Our results suggest that there is no significant long-term benefit to any stent type for the management of aortoiliac atherosclerotic disease, with a possible short-term benefit for BESs, with nearly 95% primary patency at 1 year. These findings suggest that stent selection should be primarily based on lesion characteristics and operator preference. Given the significant cost associated with CSs, their use may be best reserved for iatrogenic perforations. The lack of a clear result in our study mirrors the literature at large. These findings may be particularly useful to treating clinicians, given that they are derived from community-level, real-world data rather than randomized controlled trials with strictly defined, protocolized care.

The limitations of our study include its retrospective design and the potential for selection bias, despite attempts to ensure comparable groups for all stent types. In addition, there were a higher number of BESs deployed in our study than other stent types. The unequal distribution of stent types could contribute to the higher rates of patency observed in BESs; however, these results are largely descriptive in nature and reflect real-world use. Another limitation is that only a minority of the lesions treated in our dataset were TASC C/D, limiting our ability to make inferences on more complex lesions. Furthermore, CSs were not defined as balloon-expandable or self-expanding when coded, which could cause confounding given the distinct nature of BESs and SESs. Use of a registry such as the VQI database brings other limitations, including reliance on an external authorized user to correctly identify indications, lesion severity, and stent selection. Given the de-identified nature of the data, it is impossible to verify every detail. It is possible for details of the procedure to be missed in the predefined outcomes of this registry. In addition, the VQI dataset is limited in terms of extensive follow-up.

Based on our findings, we conclude that there is no clear benefit to any specific stent type for the treatment of aortoiliac artery disease at present. Decisions regarding stent type should be guided by TASC lesion severity, lesion characteristics, and operator preference. Further studies with real-world data are needed to guide clinicians on optimal stent selection.

## Data Availability

The datasets presented in this article are not readily available because the data underlying this study were obtained under a Data Use Agreement with the VQI Database (#3940). A request for the datasets can be made to the Arterial Research Advisory Council. Requests to access the datasets should be directed to https://www.vqi.org/data-analysis/.
