# Supplementary material for: Comparison of aortoiliac disease stenting patency using balloon-expandable, self-expanding, and covered stents
Source: Front Cardiovasc Med. 2026 May 13;13:1691067. doi: 10.3389/fcvm.2026.1691067 (PMC13212113; doi:10.3389/fcvm.2026.1691067)
Supplement: Supplementary file 1 [file Supplementaryfile1.docx]

Supplementary Material

**Supplementary Methods** Page 1

**Supplementary Figure** Page 2

**Supplementary Methods: Variable definitions**

Variable definitions used in this study were the same as described in the VQI database. Coronary artery disease was defined as any prior history of angina or myocardial infarction. Congestive heart failure was defined as asymptomatic, mild, moderate, or severe. Chronic obstructive pulmonary disease was defined as not being treated, on medications, or on home oxygen. Diabetes was defined as either diet, non-insulin-dependent medications, or insulin-dependent. Dialysis was defined as being on dialysis or having a history of transplant. Hypertension was defined as blood pressure >140/90 mmHg or a history of hypertension. Smoking was defined as either prior or current smoker.

Pre-medications were also assessed. Statins were considered pre-medications if taken at least 36 hours prior to the procedure. All other medications were defined as pre-medications if taken within 30 days prior to procedure.

## Supplementary Figures


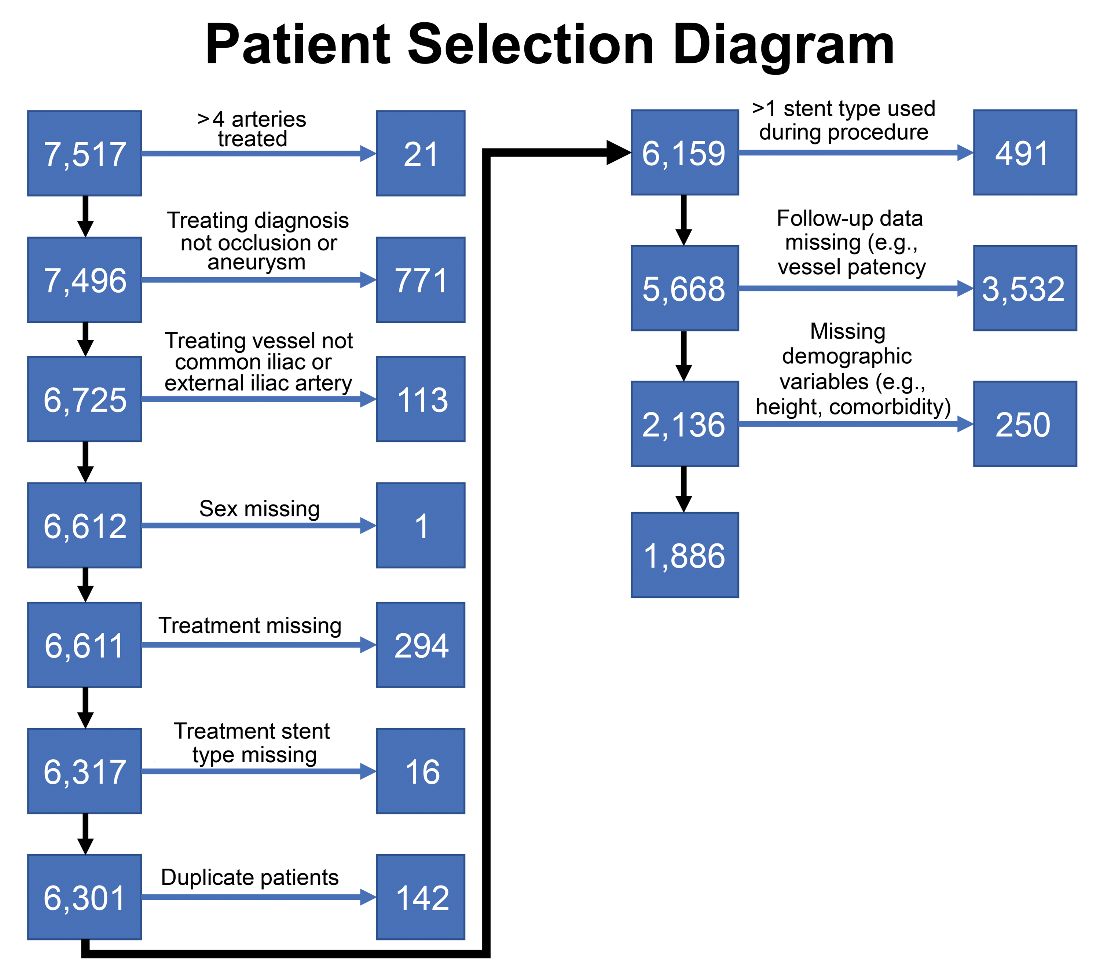


**Supplementary Figure 1.** Patient selection flow chart.

A query of the Vascular Quality Initiative database for all patients who had undergone aortoiliac intervention from January 2012 to September 2017 identified 7,517 patients. After applying exclusion criteria, 1,886 patients with a total of 2,673 lesions, were included in the study.
